# Comparison of Clinical Features and Treatment Outcome in Benign and Malignant Lacrimal Sac Tumors

**DOI:** 10.1155/2020/3545839

**Published:** 2020-01-31

**Authors:** Che-Yuan Kuo, Chieh-Chih Tsai, Shu-Ching Kao, Wen-Ming Hsu, Catherine Jui-Ling Liu

**Affiliations:** ^1^Department of Ophthalmology, Taipei Veterans General Hospital, Taipei, Taiwan; ^2^Department of Ophthalmology, School of Medicine, National Yang-Ming University, Taipei, Taiwan; ^3^Taipei Medical University-Shuang-Ho Hospital, Ministry of Health and Welfare, New Taipei City, Taiwan

## Abstract

**Purpose:**

To compare the clinical characteristics and treatment outcome between benign and malignant lacrimal sac tumors.

**Methods:**

We retrospectively reviewed the medical records of all patients with pathologically confirmed lacrimal sac lesions from 1995 to 2018 in a tertiary medical center.

**Results:**

Among 65 eligible cases, 46 (70.8%) were benign lacrimal sac tumors and 19 (29.2%) were malignant lacrimal sac tumors. Secondary malignancy from nasal or paranasal cancer accounted for 47% of malignant lacrimal sac tumors. The patient's mean age at the time of diagnosis was 60 years in the benign group and 48 years in the malignant group (*p*=0.03). The most common presenting symptoms were a palpable lump/mass and epiphora in both groups. Palpable mass extending above the medial canthal tendon was noted in 9% of the benign group and in 74% of the malignant group, respectively (*p*=0.03). The most common presenting symptoms were a palpable lump/mass and epiphora in both groups. Palpable mass extending above the medial canthal tendon was noted in 9% of the benign group and in 74% of the malignant group, respectively (*p*=0.03). The most common presenting symptoms were a palpable lump/mass and epiphora in both groups. Palpable mass extending above the medial canthal tendon was noted in 9% of the benign group and in 74% of the malignant group, respectively (*p*=0.03). The most common presenting symptoms were a palpable lump/mass and epiphora in both groups. Palpable mass extending above the medial canthal tendon was noted in 9% of the benign group and in 74% of the malignant group, respectively (

**Conclusion:**

Although benign and malignant lacrimal sac tumors may present similar initial symptoms, timely diagnosis and intervention for malignant lacrimal sac lesions are important because they tend to be infiltrating tumors with a poor outcome.

## 1. Introduction

Lacrimal sac tumors are relatively rare compared to other ocular adnexal tumors. Diagnosis of these tumors is often delayed because they are usually confused with inflammatory dacryocystitis [1–6]. Moreover, because of its location between paranasal sinus and the orbit, lacrimal sac tumor is less noticeable during its initial stage than eyelid tumor or conjunctival tumor. Most published studies mainly focused on malignant lacrimal sac tumors. In the current study, we compare the clinical characteristics and treatment outcome between benign and malignant lacrimal sac tumors.

## 2. Method

We retrospectively reviewed the medical records of all patients with pathologically confirmed lacrimal sac lesions who were treated at Taipei Veterans General Hospital between 1995 and 2018. Collected data included age, gender, initial presenting symptoms, signs, computed tomography (CT) imaging, treatment, and outcome. The clinical features and treatment outcome difference between benign and malignant lacrimal sac tumors were compared. Data were calculated using Microsoft Office Excel 2016. Significant differences between the two groups were studied using 2-tailed Student's *t*-test and Fisher's exact test. A value of *p* < 0.05 was considered statistically significant. This study was approved by the institutional review board of Taipei Veterans General Hospital and was conducted in accordance with the Declaration of Helsinki.

## 3. Result

A total of 65 lacrimal sac tumors with histopathologic confirmation were included in our study, with 46 (70.8%) benign tumors and 19 (29.2%) malignant tumors. Among malignant tumors, 10 (52.6%) were primary malignant tumors and 9 (47.4%) were of secondary origin, mostly invading from neighboring paranasal sinuses, nasal cavity, and eyelid. The most common presenting symptoms were mass and epiphora in benign lacrimal sac tumors (47% and 32%, respectively), in primary lacrimal sac malignant tumors (60% and 50%, respectively), and in secondary malignant lacrimal sac tumors (50% and 33%, respectively) (Table 1). Patients with secondary malignancy might also present with nasal obstruction, epistaxis, tinnitus, or otalgia. Bloody tears were observed in 5% of the benign group and in 20% of the malignant group (*p*=0.21). The demographic features and outcome of benign and malignant lacrimal sac tumors are shown in Table 2. There was a relative female predominance (59%) among patients with benign lacrimal tumors and male predominance (58%) among patients with malignant lacrimal sac tumors, although the difference did not reach the statistical significance (*p*=0.22). The patients' mean age at the time of diagnosis was 60.2 years in the benign group and 48 years in those with malignant lacrimal sac tumors (*p*=0.03). The rate of tumor mass extending above the medial canthal tendon was 9% in the benign group and 74% in the malignant group (*p* < 0.001). On CT imaging, primary malignant lacrimal sac tumors had a higher incidence of surrounding bone erosion (50% vs. 11%, *p* < 0.05) and presenting as infiltrative lesions (63% vs. 0%, *p* < 0.05) as compared to that of benign lacrimal sac lesions. In the malignant group, surgical resection was performed in all 19 patients, with adjuvant concurrent chemoradiotherapy in 9 cases and radiotherapy in 8 cases. The recurrence rate was significantly higher in patients with malignant tumors, as compared to the benign tumors (42% and 6%, respectively, *p*=0.001). The metastatic rate was 47% and the mortality rate was 53% in malignant lacrimal sac tumors.

In subgroup analysis for malignant tumors, the most common primary malignant lacrimal sac tumors were 3 cases of diffuse large B-cell lymphoma (DLBCL), followed by 2 cases of squamous cell carcinoma (SCC), 2 cases of lymphoepithelial carcinoma, 1 case of adenoid cystic carcinoma (ACC), 1 case of leiomyosarcoma, and 1 case of malignant melanoma. The most common secondary malignant lacrimal sac tumors were SCC (4 cases), followed by ACC (2 cases), basal cell carcinoma (1 case), mucoepidermoid carcinoma (1 case), and poorly differentiated carcinoma (1 case).  Table 3 compared the demographic features and outcome between primary and secondary malignant lacrimal sac tumors. There was a significant female predominance among primary malignant tumors and male predominance among secondary tumors (*p*=0.02). The mean age at the time of diagnosis was relatively younger in patients with primary malignant lacrimal tumors as compared to that in secondary malignant lacrimal sac tumors (43.5 vs. 51.6 years, *p*=0.07). Local recurrence rate, metastatic rate, and mortality rate were high and similar in both groups.

## 4. Discussion

Our study revealed that the initial presenting symptoms such as epiphora and a lump or swelling in the lacrimal sac area are often similar in both benign and malignant lacrimal sac lesions. However, tumor mass extending above the medial canthal tendon remained an important red flag sign suggestive of malignant lacrimal sac tumors. Besides, imaging features such as bone erosion or infiltrative lesions would also indicate the possibility of malignancy. In particular, secondary malignancy accounted for 47% of malignant lacrimal sac tumors and may present with nasal symptoms. They often infiltrated from paranasal sinus or nasal cavity to lacrimal sac and showed a poor outcome.

Because malignant lacrimal sac tumors are rare, the number of subjects recruited in the current study is limited. In the present study, we found that in patients with primary malignant lacrimal sac tumors, the mean age at the time of diagnosis was 43.5 years, which was younger than that of secondary malignant lacrimal sac tumors (51.6 years) and benign lacrimal sac tumors (60 years). Previous studies also reported that the malignant lacrimal sac tumors often occurred in the fifth decade [7–10]. No significant gender difference between benign and malignant lacrimal tumors was found in the current study. Bi et al. reported in a retrospective study of 96 cases that primary lacrimal sac tumors occurred more often in men in than women (M : F = 1.8 : 1.0), but Parmar and Rose revealed the opposite result (M : F = 1.0 : 2.75) [9, 11]. In subgroup analysis for malignant lacrimal sac tumors, we found female predominance in the primary malignant group and male predominance in the secondary malignant group. Since most of the secondary lacrimal sac tumors originated from adjacent structures, such as paranasal sinuses and nasal cavity, we hypothesized that the reason for this gender predilection may attribute to that men are traditionally more likely to develop nasopharyngeal cancer or nasal tumors than women [12].

In a review article reported by Krishna and Coupland, more than 55% of the lacrimal sac tumors were malignant [8]. However, the malignant tumors accounted for only about 30% in our study, which may be resulted from a higher percentage of benign subjects enrolled. Similar initial symptoms mimicking benign lesions and less noticeable tumor location could cause delayed diagnosis of malignant lacrimal sac tumors [13]. Moreover, because of its location between paranasal sinus and the orbit, management of lacrimal sac tumors is quite challengeable and often requires adjuvant therapy [4, 14–18]. Regarding the prognosis, El-sawy et al. proposed that the eye can be spared and visual function can be preserved with multidisciplinary therapy [14]. However, despite the aggressive treatments, 53% of the malignant cases in the present study still died of the disease. In a case series published by Parmar and Rose , 20% (3/15) of patients developed metastasis, and 20% (3/15) of patients died during the follow-up interval [11].

Based on current findings and previous studies, we will suggest that lacrimal sac malignancy should always be beard in mind in patients with epiphora and swelling/mass over medial canthal region. Comprehensive history taking including sinonasal symptoms, physical examinations, and imaging studies can be helpful in early diagnosis and the management of malignant lacrimal sac tumors to reduce the morbidity and mortality caused by malignant lacrimal sac tumors.

## Figures and Tables

**Table 1:** Initial presenting symptoms among patients with lacrimal sac lesions.

	Benign	Malignant		*p* value
		Primary	Secondary	
Lump/mass	9/19^*∗*^ (47%)	6/10 (60%)	3/6^*∗*^ (50%)	0.8
Epiphora	6/19^*∗*^ (32%)	5/10 (50%)	2/6^*∗*^ (33%)	0.61
Discharge	3/19^*∗*^ (16%)	1/10 (10%)		0.54
Pain	2/19^*∗*^ (11%)	4/10 (40%)		0.06
Bloody tears	1/19^*∗*^ (5%)	2/10 (20%)		0.21
Proptosis		2/10 (20%)		
EOM limitation		1/10 (10%)		
Headache		1/10 (10%)		
Nasal obstruction			1/6^*∗*^ (17%)	
Epistaxis			1/6^*∗*^ (17%)	
Tinnitus			1/6^*∗*^ (17%)	
Otalgia			1/6^*∗*^ (17%)	

Values are presented as *n* (%). ^*∗*^Symptom data were available on 19 patients with benign lesions, 10 patients with primary malignant lesions, and 6 patients with secondary malignant lesions.

**Table 2:** Comparison of demographic features and treatment outcomes between benign and malignant lacrimal sac tumors.

	Benign (*n* = 46)	Malignant (*n* = 19)	*p* value
Gender			
Male	19	11	0.22
Female	27	8	
Mean onset age (years)	60.2 ± 15.2	48 ± 9.3	0.03^*∗*^
Mass extending above the medial canthal tendon	4/46 (9%)	14/19 (74%)	<0.001^*∗*^
CT imaging^*∗∗*^			
Bone erosion	1/9 (11%)	8/16 (50%)	<0.05^*∗*^
Infiltrative lesion	0/9 (0%)	10/16 (63%)	<0.05^*∗*^
Treatment			
Surgical resection	38	19	
Adjuvant RT		8	
Adjuvant CCRT		9	
Local recurrence rate	3/46 (6%)	8/19 (42%)	0.001^*∗*^
Metastatic rate	0	9/19 (47%)	
Mortality rate	0	10/19 (53%)	
Mean follow-up time (months)	48.9 (0.25–128)	76.2 (3–168)	

Values are presented as *n* (%), mean ± SD or mean (range). CT, computed tomography; RT, radiotherapy; CCRT, concurrent chemoradiotherapy. ^*∗*^*p* < 0.05. ^*∗∗*^CT imaging data were available on 9 patients with benign lesions and 16 patients with malignant lesions.

**Table 3:** Comparison of demographic features and treatment outcomes between primary and secondary malignant lacrimal sac tumors.

	Primary (*n* = 10)	Secondary (*n* = 9)	*p* value
Gender			
Male	3	8	0.02^*∗*^
Female	7	1	
Mean age (years)	43.5 ± 3.1	51.6 ± 11.3	0.21
Local recurrence rate	2/10 (20%)	6/9 (67%)	0.07
Metastatic rate	5/10 (50%)	4/9 (44%)	1
Mortality rate	5/10 (50%)	5/9 (56%)	1

Values are presented as *n* (%) or mean ± SD. ^*∗*^*p* < 0.05.

## Data Availability

The clinical data used to support the findings of this study are included within the article.
